# Diagnostic Yield of Whole Exome Sequencing in Pediatric Dilated Cardiomyopathy

**DOI:** 10.3390/jcdd4030011

**Published:** 2017-08-08

**Authors:** Pamela A. Long, Jared M. Evans, Timothy M. Olson

**Affiliations:** 1 Mayo Graduate School of Biomedical Sciences, Molecular Pharmacology and Experimental Therapeutics Track, Mayo Clinic, Rochester, MN 55905, USA; pamela.long@cchmc.org; 2 Division of Biomedical Statistics and Informatics, Department of Health Sciences Research, Mayo Clinic, Rochester, MN 55905, USA; evans.jared@mayo.edu; 3 Division of Pediatric Cardiology, Department of Pediatric and Adolescent Medicine, Mayo Clinic, Rochester, MN 55905, USA; 4 Department of Cardiovascular Medicine, Mayo Clinic, Rochester, MN 55905, USA

**Keywords:** dilated cardiomyopathy, heart failure, pediatric, genetics, whole exome sequencing, mutation

## Abstract

Dilated cardiomyopathy (DCM) is a heritable, genetically heterogeneous disorder characterized by progressive heart failure. DCM typically remains clinically silent until adulthood, yet symptomatic disease can develop in childhood. We sought to identify the genetic basis of pediatric DCM in 15 sporadic and three affected-siblings cases, comprised of 21 affected children (mean age, five years) whose parents had normal echocardiograms (mean age, 39 years). Twelve underwent cardiac transplantation and five died with severe heart failure. Parent-offspring whole exome sequencing (WES) data were filtered for rare, deleterious, de novo and recessive variants. In prior work, we reported de novo mutations in *TNNT2* and *RRAGC* and compound heterozygous mutations in *ALMS1* and *TAF1A* among four cases in our cohort. Here, de novo mutations in established DCM genes—*RBM20*, *LMNA, TNNT2,* and *PRDM16*—were identified among five additional cases. The *RBM20* mutation was previously reported in familial DCM. An identical unreported *LMNA* mutation was identified in two unrelated cases, both harboring gene-specific defects in cardiomyocyte nuclear morphology. Collectively, WES had a 50% diagnostic yield in our cohort, providing an explanation for pediatric heart failure and enabling informed family planning. Research is ongoing to discover novel DCM genes among the remaining families.

## 1. Introduction

Idiopathic dilated cardiomyopathy (DCM) is a heritable, genetically heterogeneous disorder characterized by progressive degeneration of cardiac muscle that leads to heart failure. DCM is often clinically silent in childhood, with delayed diagnosis at a mean age of 45 years [1]. However, a subset of patients develops symptomatic heart failure at a young age, suggesting unique pathogenic mechanisms for DCM within the pediatric age group. In both children and adults, end-stage DCM remains the most common indication for cardiac transplantation [2,3]. While mutations identified in more than 50 DCM genes have advanced understanding of disease pathobiology [4,5], the diagnostic yield of clinical gene panel testing for DCM is only 37% [6]. Moreover, emerging data suggest that the frequency and spectrum of mutated genes underlying pediatric DCM are in fact different from those causing latent DCM [6]. Beyond traditional candidate gene and locus mapping strategies for DCM gene discovery, whole exome sequencing (WES) in parent-offspring trios provides an efficient means to discover the genetic basis of sporadic DCM, and ultimately enhance the yield of molecular diagnostics. Here, we extend our research utilizing WES for disease gene discovery in a cohort of children with DCM. De novo mutations in four established DCM genes were identified among five cases. Together with our previously reported findings [7,8,9,10], we discovered causal mutations among functionally diverse genes in 50% of pediatric DCM cases. 

## 2. Materials and Methods 

### 2.1. Study Subjects

All subjects were Mayo Clinic patients of northern European ancestry who were diagnosed with DCM at 0–17 years of age. Both prospective and retrospective recruitment of consenting families occurred between 1987 and 2014, utilizing electronic medical record search tools in later years to capture all eligible, living children. The current study was confined to sporadic and affected-sibling cases whose parents had normal screening echocardiograms. During the same time period, 19 familial cases comprised of a parent and ≥1 child with DCM were recruited. These families were excluded from this study. Each subject gave their written informed consent before they participated in the study. The study was conducted in accordance with the Declaration of Helsinki, and the protocol was approved by the Mayo Clinic Institutional Review Board (IRB #1452-00). Diagnostic criteria for DCM were left ventricular diastolic and/or systolic dimensions >95th percentile indexed for body surface area, and left ventricular ejection fraction <50% as determined by echocardiography. Genomic deoxyribonucleic acid (DNA) was isolated from peripheral blood white cells or saliva. WES and whole genome sequencing (WGS) data from 115 in-house individuals not affected with DCM were used for subtraction filtering of exome variant calls to identify true positives. 

### 2.2. WES and Bioinformatics Analysis

WES and variant annotation were performed on DNA samples from 21 children with idiopathic DCM (mean age at diagnosis, five years) and their unaffected parents (mean age at screening echocardiogram, 39 years), utilizing the Mayo Clinic Medical Genome Facility and Bioinformatics Core. The Agilent SureSelect Human All Exon capture kit (v2: DC-17, 45, 62, 68, 69, 79, 82; v4+UTRs: DC-80, 84, 86, 87; v5+UTRs: DC-70, 90, 92, 94, 95, 97, 101; Agilent) was utilized for exome capture. Paired-end 101 base pair sequencing was performed on Illumina’s HiSeq2000 platform (Illumina, San Diego, CA, USA). A quality control analysis of a comprehensive list of 55 known DCM genes in DC-82 revealed that, on average, over 96% of known DCM genes had 20X coverage. Reads were aligned to the hg19 reference genome with Novoalign (http://novocraft.com) followed by sorting and marking of duplicate reads using Picard (http://picard.sourceforge.net). Local realignment of insertions/deletions (INDELs) and base quality score recalibration were then performed using the Genome Analysis Toolkit (GATK) [11]. Single nucleotide variants (SNVs) and INDELs were called across family units simultaneously using GATK’s UnifiedGenotyper with variant quality score recalibration [12]. The resultant variant call format files were analyzed with Ingenuity^®^ Variant Analysis^TM^ software (QIAGEN, Redwood City, CA, USA). For families DC-68 and DC-80, each of the two affected children was required to carry a variant for it to pass filtering criteria. To determine rarity of variants, minor allele frequencies from three publicly available population databases were used: 1000 Genomes (1000 G, WGS data from 1092 individuals) [13]; European individuals in Exome Variant Server (EVS, WES data from 4300 individuals) [14]; or European individuals in the Exome Aggregation Consortium (ExAC, WES data from 33,370 individuals) [15]. Because *TTN* is highly tolerant of single nucleotide variation (RVIS_ExAC = 99.5%) [15] and rare missense variants are common in healthy controls, missense variants in this gene were excluded from the analyses [5]. Sanger sequencing was performed to confirm pathogenic variants, with primer pairs listed in Table S1. In silico tools were utilized to predict the biological impact of identified mutations. We employed Combined Annotation-Dependent Depletion (CADD) scores [16] for missense mutations, and probability of loss-of-function intolerance (pLI) scores for genes harboring heterozygous truncation mutations [15]. 

### 2.3. Transmission Electron Microscopy

Formalin-fixed, paraffin-embedded (FFPE) cardiac tissue procured at cardiac transplantation was available from the patients harboring de novo *LMNA* mutations. Two additional specimens served as controls. Cardiac tissue procured at the time of autopsy from a 13-year-old male who died from non-cardiac causes served as a pediatric normal control. Cardiac tissue procured at the time of cardiac transplantation from a 12-year-old female who was negative for *LMNA* variants served as a pediatric DCM control. Formalin fixed cardiac tissue was deparaffinized by standard clinical laboratory protocols. Tissues were rinsed three times in 0.1 M sodium phosphate buffer, post-fixed, and stained in 1% osmium tetroxide. Tissues were then rinsed three times in distilled water, en bloc stained in 2% aqueous uranyl acetate, and dehydrated in a graded series of ethanol followed by absolute acetone. Tissues were subsequently infiltrated and embedded in epoxy resin. Semi-thin (0.6 μm) sections for light microscopy were cut with an ultramicrotome and stained with toluidine blue. Thin sections, approximately 100 nm in thickness, were cut with an ultramicrotome, post-stained with 0.3% aqueous lead citrate, and examined in a FEI Tecnai G^2^ 12 transmission electron microscope operated at 80 kV. Digital images were captured with a Gatan Model 785 ES1000W Erlangshen side mount 4k × 2.7k CCD camera and Digital Micrograph software (Gataon, Pleasanton, CA, USA).

## 3. Results

### 3.1. WES Uncovers De Novo Mutations as a Cause for Early-Onset DCM 

**Figure 1:**
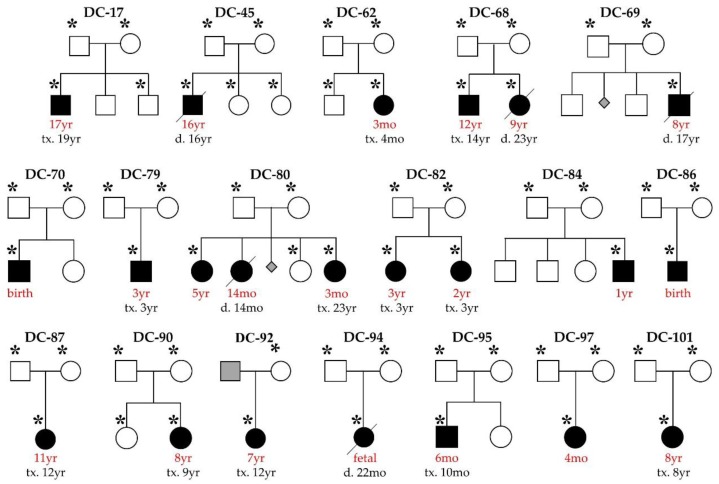
Pediatric dilated cardiomyopathy (DCM) cohort. Square, male; circle, female; black, affected; white, unaffected; grey, unknown; triangle, miscarriage; red font, age at diagnosis; slash, deceased; asterisk, whole exome sequencing (WES) performed. Causal mutations were previously reported in kindreds DC-82, DC-86, DC-87, and DC-94. These families were excluded from the current study except for determination of WES diagnostic yield.

**Figure 2:**
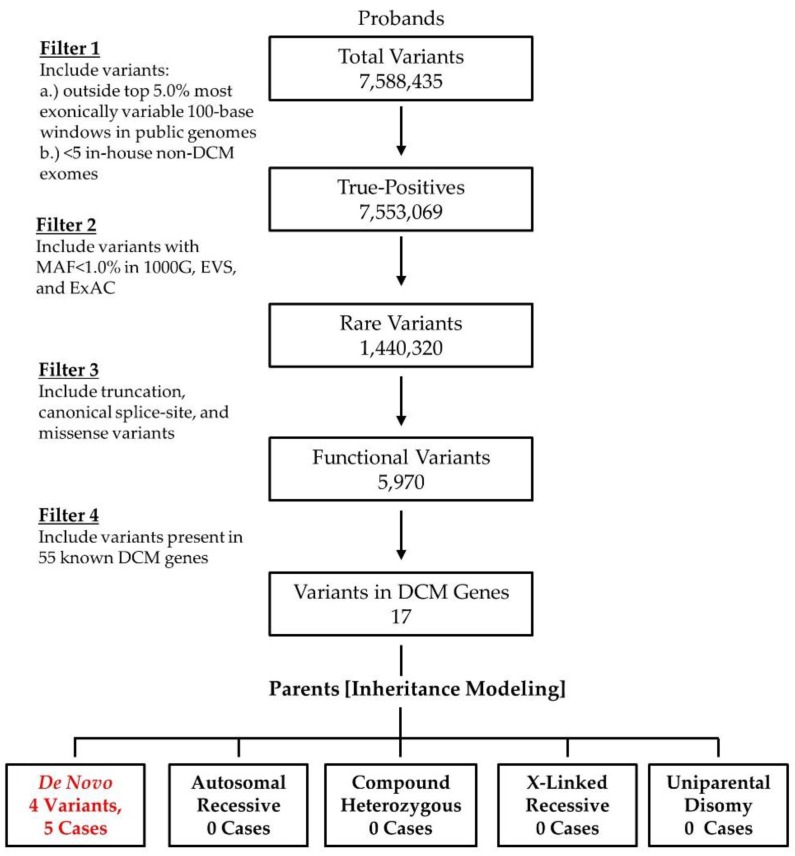
WES filtering scheme in 14 families uncovered five de novo mutations within established DCM genes.

Our pediatric DCM cohort is comprised of 18 families (Figure 1). Three cases are syndromic: DC-86 (Alström syndrome); DC-94 (facial dysmorphism, congenital cataracts, septal defects,), and; DC-69 (facial dysmorphism, cognitive impairment). Clinical DCM gene panel testing had been independently performed in five cases and was non-diagnostic in four due to absence of the disease gene on the selected panel (*ALMS1* in DC-86, *PRDM16* in DC-97) or novelty of the disease gene (*TAF1A* in DC-82, *RRAGC* in DC-94). For the current study, WES data from 14 of the previously unreported families were binned and analyzed with Ingenuity^®^ Variant Analysis^TM^ software (QIAGEN, Redwood City, CA, USA). In total, over 7 million variants were identified among cases. To isolate rare, coding variants in known DCM genes, an iterative variant filtering approach was employed (Figure 2). Variants were first filtered to retain true positives by excluding those outside the top 5% of most exonically variable 100-base windows in population-based genomes, as well as those present in five or more in-house, non-DCM controls. Next, variants were filtered for rarity, excluding those with a minor allele frequency ≥1.0% in 1000 Genomes, Europeans in EVS, or Europeans in ExAC. All missense, truncation, in-frame INDEL, and canonical splice-site variants were retained. Variants were then filtered for those occurring within 55 known DCM genes, 50 of which were included on one or more commercially available genetic testing panels (Table S2). Mutations in *ALMS1*, *RRAGC*, and *TAF1A* were excluded previously. Finally, family-specific segregation analyses were employed, comparing filtered variant data for each proband in conjunction with parental WES data. Of the 17 variants, 13 were inherited from an unaffected parent (Table S3), effectively excluding them as a monogenic basis for DCM.

**Figure 3:**
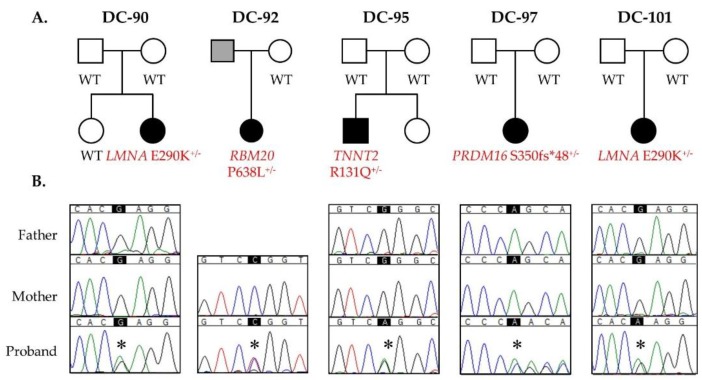
De novo mutations in *LMNA*, *RBM20*, *TNNT2*, and *PRDM16* as a cause for sporadic pediatric DCM. (**A**) Pedigrees; (**B**) Sanger sequencing confirmed each de novo mutation. Asterisks indicate position of single nucleotide alterations.

The remaining five variants within four known DCM genes all occurred as de novo germline mutations (Figure 3 and Table 1). All five children had non-syndromic DCM. The proband in DC-95, diagnosed at six months of age and transplanted at 10 months of age, harbored a missense mutation in *TNNT2* that was absent in population controls and predicted to be damaging. The proband in family DC-92, diagnosed at seven years of age and transplanted at 12 years of age, harbored a previously reported familial DCM missense mutation in *RBM20* [17] that was absent in population controls and predicted to be damaging. The proband in DC-97, diagnosed at four months of age, harbored a frameshift mutation in *PRDM16*, resulting in the addition of an anomalous peptide tail consisting of 48 residues and prematurely truncating the protein product. *PRDM16* is predicted to be highly intolerant to loss of function. The proband in DC-90, diagnosed at eight years of age and transplanted at nine years of age, and DC-101, diagnosed and transplanted at eight years of age, harbored the same missense mutation in *LMNA*, absent in population controls and predicted to be damaging. 

### 3.2. Recurrent LMNA Mutation Associated with Nuclear Inclusions

**Figure 4:**
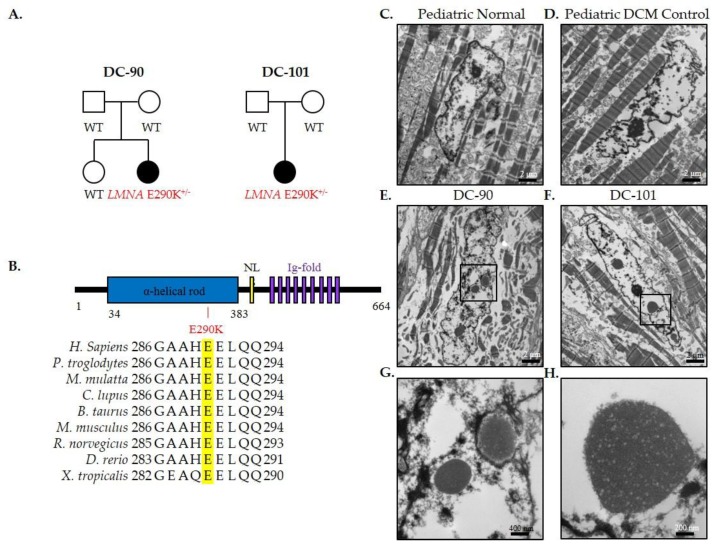
*LMNA*-associated nuclear inclusions in pediatric DCM. (**A**) Family pedigrees; (**B**) *LMNA* protein structure and conservation of E290 and surrounding residues; Electron microscopy of cardiac tissue in (**C**) pediatric normal control; (**D**) pediatric DCM control; and (**E**,**F**) patients harboring the *LMNA*-E290K mutation. Scale bars, 2 μm. Cardiomyocytes in patients with *LMNA* mutations displayed multiple nuclear blebs and inclusions, highlighted in magnified views (**G**,**H**). Scale bars 400 nm and 200 nm, respectively.

*LMNA* encodes two intermediate filaments, lamins A and C, important for maintaining the structural integrity of the nuclear lamina [18]. Explanted cardiac tissue, available from both patients harboring the identical *LMNA* E290K mutation, was analyzed by electron microscopy to determine if *LMNA*-dependent ultrastructural changes were present. Indeed, abnormal nuclear morphology was observed, characterized by nuclear blebbing and abnormal inclusions in cardiomyocyte nuclei (Figure 4E,F) that was absent in hearts of pediatric normal and pediatric DCM controls (Figure 4C,D). Inclusions appeared to originate in the nucleus and undergo successive expulsion into the cytoplasm (Figure 4G,H).

## 4. Discussion

We hypothesized that pediatric DCM is a monogenic disorder and sought to determine whether there is etiologic overlap with genes previously linked to familial DCM, more commonly diagnosed in adulthood. In this study, whole exome sequencing was performed in 14 families and 55 known DCM genes were interrogated. A total of 17 rare coding variants were identified, 13 of which were inherited from an unaffected parent, excluding their primary role in the pathogenesis of early-onset, sporadic DCM. The mean age at diagnosis of DCM and symptomatic heart failure in the children was five years, compared to a mean parental age of 39 years at the time of normal screening echocardiography. Moreover, family history of DCM in grandparents and other relatives was negative. Consequently, an autosomal dominant mode of inheritance would necessitate an extreme degree of variable penetrance. Accordingly, none of these variants were considered sufficient to cause DCM, although synergistic heterozygosity cannot be excluded, whereby paternally and maternally-inherited, hypomorphic variants from functionally related genes, for example, a known DCM gene and a novel/modifier DCM gene, could work in concert to cause DCM. Alternatively, the presence of a non-coding or structural variant on the other allele of a mutated gene would likely go undetected by WES, or a second coding variant may have gone undetected due to incomplete coverage. Testing for these potential digenic or compound heterozygous disease mechanisms would require clinical and genetic analysis of siblings to enable segregation analysis; whole genome sequencing; and/or bioinformatics tools to identify rare, predicted-damaging variants that disrupt a common biological pathway. The remaining five variants each arose as de novo events in genes with functionally diverse roles in cardiomyocytes.

*TNNT2*—*TTNT2* encodes troponin T type 2 (cardiac), mutations in which can result in aggressive, early-onset DCM [19]. *TNNT2*, part of the troponin complex, is critically important for force generation during muscle contraction, and *TNNT2*-DCM mutations have been shown to result in decreased Ca^2+^ sensitivity of force generation [20,21,22,23,24]. WES identified a R131Q in *TNNT2* located in the alpha-tropomyosin binding domain and previously identified as a likely pathogenic mutation in a six-month-old male with DCM [6].

*RBM20*—A P638L mutation was identified in *RBM20*, encoding RNA binding motif protein 20. *RBM20* is a subunit of the spliceosome that is preferentially expressed in the heart and critical in regulating alternative splicing of cardiac proteins [25,26]. The same P638L mutation has been previously identified as a cause for early-onset DCM, occurring within an arginine/serine-rich domain in exon 9 that harbors a hotspot of mutations linked to DCM [17]. While the patient’s mother did not carry the mutation, paternal DNA was unavailable to confirm that the mutation was de novo. However, the high morbidity and mortality associated with *RBM20* mutations suggests that the mutation was not inherited.

*PRDM16—PRDM16* encodes the PR domain 16 protein, a transcription factor involved in leukemogenesis, palatogenesis, neurogenesis, and brown adipose tissue differentiation [27,28,29,30]. *PRDM16* has also been shown to have a negative effect on TGF-β signaling, whereby mutations in *PRDM16* may result in disruption of cell proliferation and differentiation [27]. In zebrafish, *prdm16* has been shown to have a dominant-positive effect on cardiomyocyte proliferation, with overexpression or knockdown resulting in impaired cardiomyocyte proliferation [31]. Interestingly, missense variants in *PRDM16* have primarily been associated with DCM, while nonsense and frameshift mutations have been associated with left ventricular non-compaction (LVNC) [31], but our patient with a S350fs*48 frameshift mutation had DCM with mild features of LVNC.

*LMNA*—Lamins A and C are protein products encoded by a single gene, *LMNA*, which arise through alternative splicing [32,33]. Lamins are intermediate filaments that are important in maintaining the structural integrity of the nuclear lamina, and are involved in many cellular processes including epigenetics, chromatin organization, DNA replication, transcription, and DNA repair [18]. *LMNA* is ubiquitously expressed and mutations pleiotropic effects cause Charcot-Marie-Tooth disease, Hutchinson-Gildford progeria, limb-girdle muscular dystrophy, Emery-Dreifuss muscular dystrophy, and DCM with conduction system disease [34]. The E290K mutation has been classified as a variant of unknown significance (VUS)-favor pathogenic in a 15-year-old female [6]. However, we observed the identical mutation to arise de novo in two unrelated patients with early-onset DCM necessitating cardiac transplantation, providing compelling evidence for causality. These cases are unusual for laminopathy-mediated DCM, which rarely occurs before the age of 20 and is typically preceded by cardiac conduction system disease, which was absent in both of our patients [6,35]. The rapid development of myocardial disease could be due to unidentified modifier genes in each case, yet the veracity of this genotype-phenotype association is compelling insofar as both children had the same *LMNA* mutation and nearly identical clinical courses. Electron microscopy revealed abnormal nuclear blebbing and inclusions in both patients that was absent in pediatric normal and DCM controls (Figure 4). These findings are similar to reports describing abnormal nuclear shape, including nuclear blebbing and dispersal of DNA into the cytoplasm, in human and mouse tissue with laminopathies [36], and further expand the spectrum of previously described DCM gene-specific ultrastructural derangements [10,37,38,39].

## 5. Conclusions

An in-depth analysis of WES data for rare coding and splice-site variants within known DCM genes identified pathogenic de novo mutations in five pediatric cases. These findings indicate that sporadic and familial DCM are in fact genetically similar in a subset of cases, yet the penetrance of these mutations among functionally diverse genes differs. Modifier genes could account for young age at development of symptomatic heart failure, as we had reported in another sporadic pediatric DCM case [8]. The current study together with prior research on our pediatric cohort demonstrate the dual value of WES over gene panels for comprehensive in silico analysis of known DCM genes, including rare and syndromic genes (*ALMS1* and *PRDM16*), and the discovery of novel genes (*RRAGC* and *TAF1A*). Notwithstanding this, the overall gene coverage in WES is excellent yet imperfect in comparison to gene-targeted testing. When combined with previous findings in our 18-family pediatric DCM cohort (Figure 1 and Table 1) [7,8,9,10], the mutation detection rate was 39% for established DCM genes, similar to the yield of 37% for standard gene panel testing [6]. With the inclusion of *RRAGC* and *TAF1A*, the two new disease genes we discovered by WES [9,10], the detection rate increased to 50%. Disclosure of genetic findings has provided an explanation for heart failure in these cases and informed family counseling. Specifically, DCM recurrence risk is <1% in siblings of probands with de novo mutations, but 50% in offspring of these probands. In contrast, recessive mutations confer 25% and 0% risk of recurrence in siblings and offspring, respectively. In the remaining nine families who lack a plausible pathogenic variant(s) among known DCM genes, efforts are ongoing to discover additional novel pediatric DCM genes and ultimately expand the utility of clinical genetic testing. 

## Figures and Tables

**Table 1:** Mutations identified in DCM cohort.

Family	Gene Symbol	Inheritance	Transcript Variant	Protein Variant	CADD or pLI Score (%ile)	ExAC MAF (Eur)	Gene Ontology
DC-82 *	*TAF1A*	CH	c.251T>C c.1021G>A NM_005681.3	p.L84S p.G341R	27.2 (85) 34.0 (99)	0.007% 0.004%	rRNA transcription
DC-86 *	*ALMS1*	CH	c.4156dupA c.6436C>T NM_015120.4	p.T1386fs*15 p.R2146*	pLI = n/a	0.001% 0.001%	Formation/maintenance of primary cilia
DC-87 *	*TNNT2*	De novo	c.421C>T NM_001001430.2	p.R141W	33.0 (>95)	--	Sarcomeric force generation
DC-90	*LMNA*	De novo	c.868G>A NM_170707.3	p.E290K	35.0 (>99)	--	Nuclear lamina
DC-92	*RBM20*	Presumed de novo	c.1913C>T NM_001134363.2	p.P638L	28.0 (90)	--	Spliceosome
DC-94 *	*RRAGC*	De novo	c.224C>A NM_022157.3	p.S75Y	27.2 (85)	--	mTORC1 activation
DC-95	*TNNT2*	De novo	c.392G>A NM_001001430.2	p.R131Q	33.0 (>95)	--	Sarcomeric force generation
DC-97	*PRDM16*	De novo	c.1047dupC NM_022114.3	p.S350fs*48	pLI = 1.00 (100)	--	Transcriptional regulation
DC-101	*LMNA*	De novo	c.868G>A NM_170707.3	p.E290K	35.0 (>99)	--	Nuclear lamina

--, not reported; *ALMS1*, centrosome and basal body associated protein; CH, compound heterozygous; *LMNA*, Lamin A/C; pLI, probability of loss-of-function intolerance; MAF, minor allele frequency; n/a, not applicable (recessive disorder caused by compound heterozygous truncating mutations); *PRDM16*, PR domain containing protein 16; *RBM20*, RNA-binding motif protein 20; *RRAGC*, Ras-related GTP-binding protein C; *TAF1A*, TATA box-binding protein-associated factor 1A; *TNNT2*, troponin T2, cardiac. * previously reported mutation [7,8,9,10].

## Supplementary Materials

The following are available online http://www.mdpi.com/2308-3425/4/3/11/s1: Tables S1–S3.
