# Supplementary material for: Diagnostic Yield of Whole Exome Sequencing in Pediatric Dilated Cardiomyopathy
Source: J Cardiovasc Dev Dis. 2017 Aug 8;4(3):11. doi: 10.3390/jcdd4030011 (PMC5715713; doi:10.3390/jcdd4030011)
Supplement: Supplementary file 1 [file jcdd-04-00011-s001.pdf]

Supplementary Appendix

Table S1. Primer pairs utilized for Sanger sequencing verification.

Primer	Sequence (5'-->3')	Annealing Temperature (^{O}C)
LMNA4 E290K F	TCCACCCCTCCCAGTCACC	62
LMNA4 E290K R	CCCCTTGTCTAACTGAAACCT	
PRDM16 350fs*48 F	CACCACAGCTGAAAACGCTT	60
PRDM16 350fs*48 R	CGGCAGAGATGTCAGAACCT	
RBM20 R636S F	AGAGTTGGGAGTTAAGAGTGTA	62
RBM20 R636S R	GCTGCTGCTTCAGATACTTGT	
TNNT2 R131Q F	GGCACCATTGCTTCAAGACT	60
TNNT2 R131Q R	CTCAAGTGATCTACCCGCCTT	

Table S2. Genetic causes of DCM.

Gene	Inheritance	Allelic Disorders	Partners Healthcare Panel	$GeneD_x$ Panel	Invitae
ABCC9	AD	Atrial fibrillation; hypertrichotic osteochondrodysplasia	X	X	X
ACTC1	AD	Atrial septal defect; hypertrophic/restrictive cardiomyopathy; left ventricular noncompaction	X	X	X
ACTN2	AD	Hypertrophic cardiomyopathy; left ventricular noncompaction	X	X	X
ALPK3	AR	Hypertrophic cardiomyopathy			
ANKRD1	Unknown	Hypertrophic cardiomyopathy	X	X	X
BAG3	AD	Myofibrillar myopathy	X	X	X
CAV3	AD	Long QT syndrome; distal myopathy; limb-girdle muscular dystrophy; rippling muscle disease	X	X	X
CHRM2	AD	None	X		X
CRYAB	AD/AR	Cataract; myofibrillar myopathy	X	X	X
CSRP3	AD	Hypertrophic cardiomyopathy	X	X	X
DES	AD/AR	Limb-girdle muscular dystrophy; myofibrillar myopathy; scapuloperoneal syndrome	X	X	X
DMD	XL	Duchenne/Becker muscular dystrophy	X	X	X
DOLK	AR	Congenital disorder of glycosylation	X		X
DSC2	AD	Arrhythmogenic right ventricular dysplasia	X	X	X
DSG2	AD	Arrhythmogenic right ventricular dysplasia	X	X	X
DSP	AD/AR	Arrhythmogenic right ventricular dysplasia; Carvajal syndrome; epidermolysis bullosa; keratosis palmoplantaris striata; skin fragility-wooly hair syndrome	X	X	X
EMD	XL	Emery-Dreifuss muscular dystrophy	X	X	X
EYA4	AD	Sensorineural hearing loss			X
FHL2	Unknown	None	X		X
FKTN	AR	Dystroglycanopathy muscular dystrophy			X
GATAD1	AR	None	X	X	X
ILK	Unknown	None	X	X	X
LAMA4	Unknown	None	X	X	X
LAMP2	XL	Danon disease	X	X	X
LDB3	AD	Hypertrophic cardiomyopathy; left ventricular noncompaction; myofibrillar myopathy	X	X	X
LMNA	AD/AR	Charcot-Marie-Tooth disease; Emery-Dreifuss muscular dystrophy; familial partial lipodystrophy; heart-hand syndrome; Hutchinson-Gilford progeria syndrome, limb-girdle muscular dystrophy; left ventricular noncompaction; Malouf syndrome; Mandibuloacral dysplasia; restrictive cardiomyopathy	X	X	X
MURC	AD	None	X		
MYBPC3	AD	Hypertrophic cardiomyopathy; left ventricular noncompaction	X	X	X
MYH6	AD	Atrial septal defect; hypertrophic cardiomyopathy	X		X
MYH7	AD	Hypertrophic/restrictive cardiomyopathy; left ventricular noncompaction; myosin storage myopathy; scapuloperoneal syndrome	X	X	X
MYPN	AD	Hypertrophic/restrictive cardiomyopathy	X	X	X
NEBL	Unknown	Endocardial fibroelastosis	X	X	X

Table S2. Genetic causes of DCM (continued).

Gene	Inheritance	Allelic Disorders	Partners Healthcare Panel	GeneD$_x$ Panel	Invitae
NEXN	Unknown	Hypertrophic cardiomyopathy	X	X	X
PDLIM3	Unknown	None	X	X	X
PKP2	AD	Arrhythmogenic right ventricular dysplasia	X	X	X
PLEKHM2	AR	Left ventricular noncompaction			X
PLN	AD	Hypertrophic cardiomyopathy	X	X	X
PRDM16	AD	Left ventricular noncompaction	X		X
PSEN1	AD	Alzheimer disease; familial acne inversa; pick disease			
PSEN2	Unknown	Alzheimer disease			
RAF1	AD	LEOPARD syndrome; Noonan syndrome		X	X
RBM20	AD	None	X	X	X
SCN5A	AD	Atrial fibrillation; Brugada syndrome; long QT syndrome; sick sinus syndrome; ventricular fibrillation	X	X	X
SGCD	AD/AR	Limb-girdle muscular dystrophy	X	X	X
SYNE1	AD	Emory-Dreifuss muscular dystrophy; spinocerebellar ataxia			
TAZ	XL	Barth syndrome	X	X	X
TCAP	AR	Hypertrophic cardiomyopathy; limb-girdle muscular dystrophy	X	X	X
TNNC1	AD	Hypertrophic cardiomyopathy	X	X	X
TNNI3	AD/AR	Hypertrophic/restrictive cardiomyopathy	X	X	X
TNNI3K	AD	Atrial tacharrhythmia; conduction system disease			
TNNT2	AD	Hypertrophic/restrictive; left ventricular noncompaction	X	X	X
TPM1	AD	Hypertrophic cardiomyopathy; left ventricular noncompaction	X	X	X
TMPO	Unknown	None		X	X
TTN	AD	Hypertrophic cardiomyopathy; limb-girdle muscular dystrophy; myopathy; tibial muscular dystrophy	X	X	X
VCL	AD	Hypertrophic cardiomyopathy	X	X	X

AD, autosomal dominant; AR, autosomal recessive; XL, X-linked.

Table S3. Inherited, non-segregating variants in sporadic DCM.

Family	Gene Symbol	Inheritance	Transcript Variant	Protein Variant	CADD Score (%ile)	ExAC MAF (Eur)	Gene Ontology
**DC-45**	*CAV3*	Paternal*	c.233C>T NM_033337.2	p.T78M	23.7 (65)	0.41%	Scaffolding protein
**DC-62**	*LAMA4*	Paternal	c.227C>T NM_001105206.2	p.S76L	18.95 (35)	--	Extracellular matrix
	LDB3	Paternal*	c.493C>T NM_007078.2	p.R165W	10.7 (20)	0.01%	Z-disk
**DC-68**	*FHL2*	Maternal	c.725G>A NM_001450.3	p.R242Q	33 (>95)	0.00%	Protein-protein interaction
**DC-70**	*MYBPC3*	Maternal	c.2497G>A NM_000256.3	p.A833T	32 (95)	0.27%	Sarcomeric cytoskeleton
**DC-79**	*SYNE1*	Paternal	c.11187G>T NM_182961.3	p.K3729N	21.6 (45)	0.62%	Nuclear membrane
	MYBPC3	Paternal	c.977G>A NM_000256.3	p.R326Q	24 (65)	0.85%	Sarcomeric cytoskeleton
**DC-80**	*TMEM43*	Paternal*	c.1061G>C NM_024334.2	p.C354S	22.7 (55)	0.08%	Nuclear membrane
	ACTC1	Maternal*	c.382A>G NM_005159.4	p.T128A	23.3 (55)	--	Sarcomere force generation
**DC-92**	*PKP2*	Paternal or *de novo*	c.1592T>G NM_004572.3	p.I531S	25.6 (80)	0.77%	Desmosome
	DSC2	Paternal or *de novo*	c.1729A>G NM_024422.4	p.I577V	<10 (<2)	0.06%	Desmosome
**DC-95**	*SCN5A*	Paternal	c.488C>T NM_198056.2	p.T163I	20.4 (40)	--	Voltage-gated Na channel
	MYH6	Paternal	c.3010G>T NM_002471.3	p.A1004S	22.9 (50)	0.11%	Sarcomere force generation

--, not reported; *variant is inherited by both affected and unaffected sibling(s); *ACTC1*, actin, alpha, cardiac muscle 1; *CAV3*, caveolin 3; *DSC2*, desmocollin 2; *FHL2*, four and a half LIM domains 2; *LAMA4*, laminin subunit alpha 4; *LDB3*, LIM domain binding 3; *MYBPC3*, myosin binding protein C, cardiac; *MYH6*, myosin heavy chain 6; *PKP2*, plakophilin 2; *SCN5A*, sodium voltage-gated channel alpha subunit 5; *SYNE1*, spectrin repeat containing nuclear envelope protein 1; *TMEM43*, transmembrane protein 43.
